# Computed cardiopulmonography: Effects of physical characteristics on lung parameter estimates

**DOI:** 10.1113/EP092423

**Published:** 2025-07-17

**Authors:** Asma Alamoudi, Dominic Sandhu, Thomas D. E. Bithell, Nicholas M. J. Smith, Graham Richmond, Lorenzo S. Petralia, Snapper Magor‐Elliott, Haopeng Xu, Nick P. Talbot, Grant A. D. Ritchie, Nayia Petousi, Peter A. Robbins

**Affiliations:** ^1^ Department of Physiology, Anatomy & Genetics University of Oxford Oxford UK; ^2^ Department of Respiratory Care Prince Sultan Military College of Health Sciences Dhahran Saudi Arabia; ^3^ Department of Chemistry University of Oxford Oxford UK; ^4^ Institute for Breath Research University of Innsbruck Innsbruck Austria; ^5^ Nuffield Department of Medicine University of Oxford Oxford UK

**Keywords:** airways disease, gas exchange, lung function

## Abstract

Computed cardiopulmonography (CCP) is a technique that measures lung volumes (functional residual capacity and deadspace) together with novel parameters reflecting lung inhomogeneities (non‐uniformities in lung inflation and deadspace path length). First, highly precise measurements of gas exchange are made during a nitrogen washout with a purpose‐built molecular flow sensor. Second, an individual's lung physiology is then described by personalising the parameters of a bespoke cardio‐respiratory model obtained by fitting the model to the data. The present study examines the effects of participants’ physical characteristics on these parameter values, and from this also provides preliminary estimates for normal ranges. Data from 92 healthy individuals (27% female, age 40 ± 19 (mean ± SD) years, height 1.75 ± 0.09 m, mass 74 ± 14 kg) were used. A prediction equation for each CCP parameter was written as: *y* = α + βln(age) + γln(height) + δln(BMI) + ε(is_Female) + error, where BMI is body mass index. Non‐significant terms (*P *> 0.1) were removed sequentially to identify just the significant characteristics. Physical characteristics exerted a large influence on volume‐related CCP parameters. In contrast, only age had a significant influence on inhomogeneity‐related CCP parameters. The prediction equations, together with their mean squared errors, were used to calculate *z*‐scores for CCP data from three previously published studies in asthma, chronic obstructive pulmonary disease, and early cystic fibrosis. Values for these *z*‐scores often lay beyond those commonly used to define a normal range (±1.65). In conclusion, reference values for inhomogeneity‐based CCP parameters may only need correcting for age, and often appear as abnormal in airways disease.

## Abstract (highlights)

- **What is the central question of this study?**
  Computed cardiopulmonography (CCP) is a technique that measures lung volumes together with novel parameters reflecting inhomogeneities in lung structure/function: what is the effect of a healthy person's physical characteristics on these parameters?
- **What is the main finding and its importance?**
  CCP parameters relating to inhomogeneity were influenced only by age, and not by height, body mass index or sex. Based on these data, *z*‐scores calculated for CCP parameters in patients with airways disease were commonly abnormal, suggesting CCP may be more sensitive than the established clinical techniques for detecting airways disease.

## 1 INTRODUCTION

Computed cardiopulmonography (CCP) is an experimental technique for obtaining patient‐specific values for a number of physiological parameters relating to lung function and to the circulation. CCP has two core components. The first is experimental, and is based on obtaining highly precise, highly time‐resolved measurements of respired gas exchange using laser absorption spectroscopy (Ciaffoni et al., 2016). The second component involves fitting a bespoke model of the lung, circulation and body gas stores to these highly time‐resolved measurements of gas exchange in order to obtain model parameters that are specific to the patient (Magor‐Elliott et al., 2021; Mountain et al., 2018; O'Neill & Robbins, 2017).

The parameters estimated by CCP include well recognised ones, such as functional residual capacity (FRC) (with CCP, this is the lung gas volume that communicates directly with the atmosphere) and anatomical deadspace (*V* d). However, CCP also estimates novel parameters that reflect certain inhomogeneities in the lung, such as the standard deviation for the standardised deadspace (σ*V* d) and the standard deviation for the natural logarithm of the standardised lung compliance (σlnCl). Effectively, σ*V* d reflects the degree of non‐uniformity in deadspace path length between the mouth and the different gas‐exchanging lung units, and σlnCl reflects the degree of non‐uniformity of inflation and deflation between the different gas‐exchanging lung units during breathing. Preliminary studies to explore the value of these inhomogeneity parameters have been conducted in patients with asthma (Smith et al., 2020), chronic obstructive pulmonary disease (COPD) (Smith et al., 2022), cystic fibrosis (Sandhu et al., 2023) and in patients post‐COVID‐19 (Magor‐Elliott et al., 2022).

One difficulty with interpreting these studies has been the absence of an understanding of the way in which the physical characteristics of a participant can influence the parameter values obtained, and indeed the general absence of any normal ranges for parameters based on such an understanding. While parameter values from a patient group can be compared with those from a healthy control group to determine whether the groups are significantly different, this does not solve the problem of whether a value for a particular patient lies within the normal range. The present study sought to address these issues by studying participants with a range of differing physical characteristics who were otherwise healthy and free from respiratory disease. The overall null hypotheses under test were that the values of the CCP parameters were unaffected by the physical characteristics of age, height, body‐mass index and sex.

For each CCP parameter, regression was used to identify whether particular physical characteristics had a significant influence on the parameters' value. The effects were visualised graphically for each significant characteristic in turn by controlling for the influence of all other significant characteristics. The regression equations, together with their residuals, were also used to provide a set of very preliminary estimates for the normal ranges for each parameter associated with a given set of physical characteristics. Any individual's CCP parameter values could then be given associated *z*‐scores, which express the number of standard deviations by which a particular parameter value differs from the expected parameter value. By way of illustration, an initial set of *z*‐scores were calculated for CCP parameters obtained from preliminary studies of CCP in asthma, COPD and cystic fibrosis (Sandhu et al., 2023; Smith et al., 2020, 2022).

## 2 METHODS

### 2.1 Participants

Datasets were obtained from 106 participants, aged 19–80. Thirty‐one of these datasets were taken from previous publications (Magor‐Elliott et al., 2022; Sandhu et al., 2023). The remainder of the datasets (75) were previously unpublished, and of these, 21 were obtained specifically to broaden the overall age distribution. We did not exclude participants on the grounds of obesity, but participants were all otherwise healthy and had no history of respiratory disease. None had ever smoked.

All experimental work was carried out in accordance with the general principles of the *Declaration of Helsinki*. All participants gave written informed consent prior to study. Ethics approval for the study was obtained from the South Central Oxford A Research Ethics Committee (reference number 17/SC/0172).

### 2.2 Computed cardiopulmonography

Each participant was studied using CCP with a protocol that lasted ∼12 min. The protocol involved breathing normally through a mouthpiece while wearing a noseclip. Air was administered as the inspired gas for the initial ∼7 min of the protocol, and pure oxygen was administered for the final ∼5 min of the protocol.

During the protocol, gas exchange was measured in a highly precise, highly time‐resolved manner using a purpose‐built molecular flow sensor (Ciaffoni et al., 2016). The flow sensor analyses the CO_2_, O_2_ and water vapour (the balance gas is assumed to be N_2_ and calculated by subtraction) every 10 ms using laser absorption spectroscopy within the main gas stream. The advantages of conducting the analysis within the main gas stream are that there are no delays introduced by sampling catheters and that the analyses are all conducted under the same physicochemical conditions as pertain to the overall gas flow. In turn this makes it possible to calculate the viscosity and density for the respired gas, which along with measurements every 10 ms of temperature and absolute pressure, enables a highly accurate measurement of total flow to be made using pneumotachography. The accuracy of this measurement is such that it makes it possible to use the 10‐ms experimental data to drive a computational model of the lungs for many minutes without the accumulation of a significant integration error.

Following data collection, the second component of CCP involved fitting a computational model of the lungs (including inhomogeneities) (Mountain et al., 2018), blood (O'Neill & Robbins, 2017) and body gas stores (Magor‐Elliott et al., 2021) to the data. This model is composed of: (i) body tissues that consume O_2_ and produce CO_2_ via metabolism; (ii) a model of the blood that enables exchange of O_2_, CO_2_ and other gases between blood and the tissues; (iii) a model of the circulation that allows transport of O_2_, CO_2_ and other gases by blood between the tissues and the lungs; and (iv) a model of the lung that allows for the exchange of O_2_, CO_2_ and other gases between the alveolar gas and the blood and also for the ventilation of the lungs. The governing equations of the model are all based on the conservation of matter, and, for the reaction of O_2_ and CO_2_ with blood, on the law of mass action. The behaviour of this model is governed by a number of physiological parameters, and CCP, by fitting the model to the data, ascribes personalised values to these parameters that then represent the particular individual's (patho)physiology. In practice, this was achieved by driving the computational model with the experimentally recorded respiratory flow and inspired gas compositions and allowing the model to predict the expired gas compositions (which, when combined with the experimentally recorded expiratory flow, provide the expiratory molar flows for each gas species). In general, these model predictions for the molar flows during expiration for the different gas species will not match those that were recorded from the individual during the study. However, when the model is run within a non‐linear least‐squares optimisation routine, the parameters of the model can be adjusted by the routine in order to minimise the error between the measured and the modelled molar gas flows during expiration, and in this way provide estimates for the parameters that are person specific and reflect their anatomy and physiology.

**FIGURE 1:**
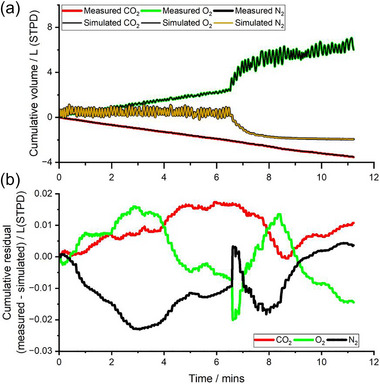
Example record for the fit of the model's gas exchange for CO_2_, O_2_ and N_2_ to the experimentally recorded gas exchange for CO_2_, O_2_ and N_2_. (a) Cumulative volume at mouth. (b) Cumulative error in gas exchange (measured minus simulated).

**FIGURE 2:**
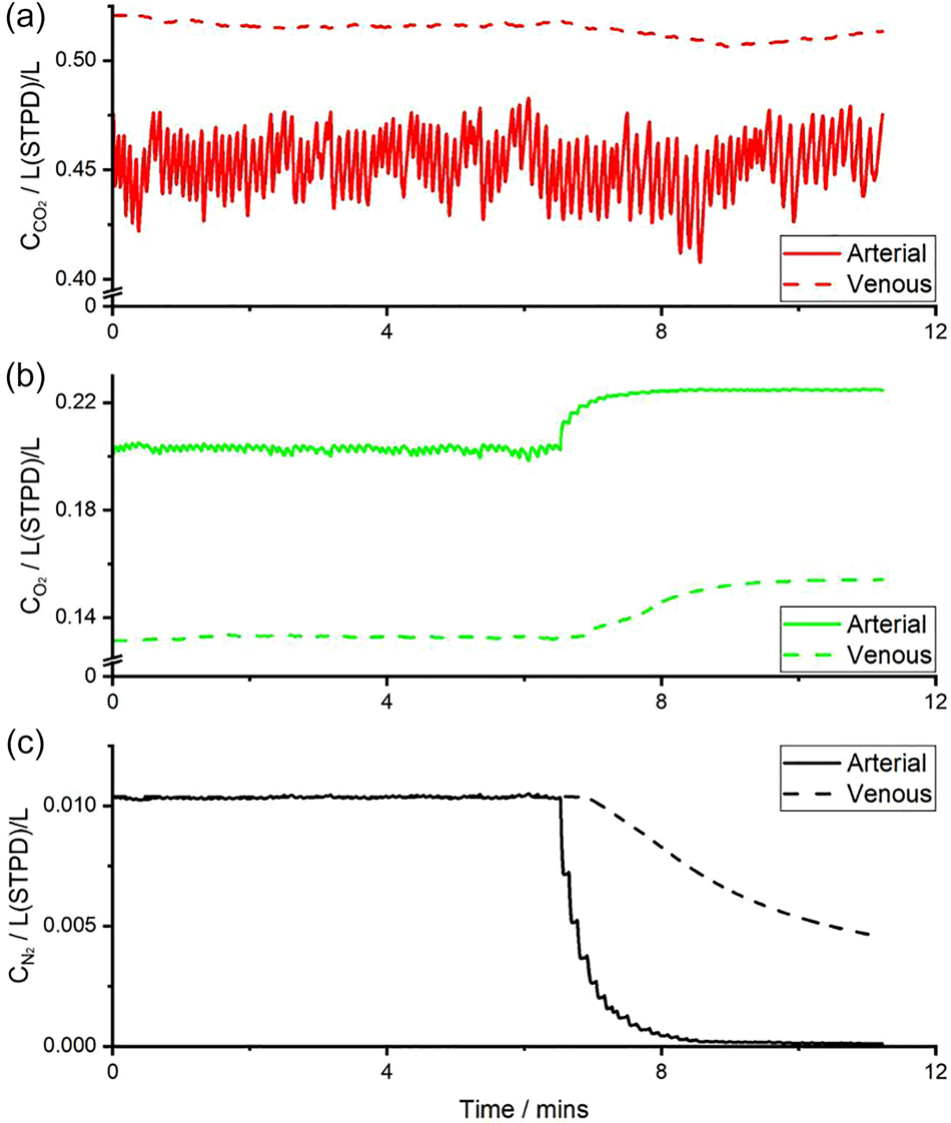
Example set of calculated blood gas contents from the model for systemic arterial and mixed venous blood. (a) CO_2_ ($C_{CO_{2}}$), (b) O_2_ ($C_{O_{2}}$), (c) N_2_ ($C_{N_{2}}$).

An example record of the tidal flows for O_2_, CO_2_ and N_2_ during the 7 min of breathing air followed by the 5 min of breathing pure O_2_ is shown in Figure 1. The increase in the tidal flow of O_2_ to and fro that occurs following the onset of breathing pure O_2_ is obvious, as is the corresponding loss in the tidal flow of N_2_. Figure 1a illustrates both the experimental data for gas exchange and the gas exchange generated by the model after the parameters of the model had been optimised to minimise the degree to which the model output deviates from the experimental data. The residual error between the two is scarcely visible, and only becomes apparent if the cumulative residuals are plotted at much higher resolution as in Figure 1b. Figure 2 illustrates the systemic arterial and mixed venous concentrations for CO_2_, O_2_ and N_2_ that are generated within the model for the data and model fit associated with Figure 1.

Table 1 lists all the parameters that are obtained by fitting the model to the gas exchange data. Although all parameters are estimated simultaneously through non‐linear regression and will to some extent influence each other's values, it is nevertheless the case that the values obtained for the different parameters will tend to be influenced by different features within the gas exchange data. The ideal $P_{CO_{2}}$ (${\mathrm{Pi}}_{{\mathrm{CO}}_{2}}$) reflects the optimal loading of the lung and body gas stores with CO_2_ at the start of the simulation. The metabolic O_2_ consumption (${\dot{V}}_{O_{2}}$) and respiratory quotient (*R*) parameters are affected predominantly by the net O_2_ and CO_2_ exchange at the mouth, but they will not in general be identical because there will usually also be some accumulation or depletion of CO_2_ and O_2_ within the lung and body gas stores. For the airway parameters, end‐expiratory alveolar volume (*V* a) is essentially determined by the amount of N_2_ that is washed out from the lung during the period of breathing pure O_2_. σlnCl is dominated by the time course of the N_2_ washout. If compliance were completely evenly distributed across the lung, then the time course of the washout would be close to mono‐exponential and the value for σlnCl would be small. As distributions for compliance become progressively less even, the time course for the N_2_ washout deviates further from a mono‐exponential form and the corresponding values for σlnCl become progressively larger. *V* d is essentially determined by the volume of fresh gas necessary to generate phases 1 and 2 of the expirogram (plots of $P_{CO_{2}}$, $P_{O_{2}}$ and $P_{N_{2}}$ against exhaled volume) correctly. σ*V* d is predominately determined by the spread of the phase 2 response. If σ*V* d were zero, then all alveolar units would have the same deadspace and phase 2 of the expirogram would be very abrupt (close to square) because the alveolar gas from all the alveolar units would arrive at the mouth at about the same time.

**TABLE 1:** Parameters reported following the modelling fitting process.

Parameters estimated within the fitting process	
${\mathrm{Pi}}_{{\mathrm{CO}}_{2}}$ (kPa)	Ideal $P_{CO_{2}}$ at initialisation of model
${\dot{V}}_{O_{2}}$ / L (STPD)/min	Oxygen consumption
*R*	Respiratory quotient
*V* a/ L (BTPS)	Alveolar volume at functional residual capacity
σlnCl	Standard deviation for the natural logarithm of the standardised lung compliance
*V* d_e_ / L(BTPS)	Deadspace volume at functional residual capacity
σ*V* d	Standard deviation for the standardised deadspace
Cvd	Fractional expansion of deadspace relative to fractional expansion of alveolar space
Parameters derived from estimated parameters	
FRC / L (BTPS)	Functional residual capacity (FRC = *V* d_e_ + *V* a)
*V* d / L (BTPS)	End‐inspiratory deadspace which equals *V* d_e_ plus an amount by which the deadspace expands following a standard inspiration, based on the value for Cvd

One complication is that *V* d tends to vary with tidal volume. We actually fit an end‐expiratory value for *V* d (*V* d_e_) together with a deadspace compliance term (Cvd) as an auxiliary parameter to reflect the degree to which *V* d rises above *V* d_e_ during inspiration. In order to keep the volumes as consistent with prior work as possible, rather than report the parameters *V* a and *V* d_e_ directly, we instead report functional residual capacity, FRC (which equals *V* a + *V* d_e_) and an end‐inspiratory value for *V* d (which equals *V* d_e_ plus an amount by which the deadspace expands following a standard inspiration, based on the value for Cvd).

In addition to the anatomical deadspace, there is an additional ∼80 mL of external apparatus deadspace between the mouth and the measurement plane of the molecular flow sensor. A geometric model of this deadspace, together with a geometric model of the upper airway of an individual (taken from a magnetic resonance imaging study), was provided to a commercial company (TotalSim, Towcester, UK) to simulate flow through this using computational fluid dynamics in order to determine the dispersion between the mouth and the measurement plane of the instrument. The external deadspace together with measured dispersion induced within it was then added as a component of the overall CCP model.

Most of the modelling software was written in MATLAB (R2022a, Mathworks Inc, Natick, MA), but with two of the more computationally intensive functions written in C++. The MATLAB non‐linear least squares estimation routine used for the main model was lsqnonlin. The computation of the individual elements of the Jacobean matrix for each iteration of the routine was undertaken in parallel. The estimation process was run in batch mode on a single (multicore) node on one of the University's Advanced Research Computing clusters and took around 1 h to complete.

### 2.3 Statistical analysis

Prior to undertaking any statistical analysis, each dataset was checked to determine the absolute value for the nitrogen exchange during the air breathing phase of the protocol. If this absolute value exceeded 60 mL/min, then the dataset was excluded on the grounds that it was likely there was a small leak either through the noseclip or around the mouthpiece.

Following quality control, a multiple linear regression was conducted to determine which physical characteristics had a significant influence on the model parameters. The form of the linear relationship used was:

(1) $$y=\alpha +\beta \ln\left(\mathrm{age}\right)+\gamma \ln\left(\mathrm{height}\right)+\delta \ln\left(\mathrm{BMI}\right)+\varepsilon \left(\mathrm{is}\_\mathrm{Female}\right)+\mathrm{error},$$

where *y* is the observed value for either a CCP parameter or its log transform; α, β, γ, δ and ε are coefficients of the linear regression; ln() denotes the natural logarithm of the value in parentheses; and BMI is body mass index. The use of log transforms for age and height followed the practice of the Global Lung Initiative for their reference equations for static lung volumes (Hall et al., 2021). The log transform for BMI was based on data from Jones and Nzekwu (2006) for lung volumes. Predictors were removed sequentially, least significant first, until the only ones that remained were associated with a *P* < 0.1. The CCP parameters studied in this manner were FRC, *V* d, σ*V* d and σlnCl. In addition to conducting the regression using the actual values of these parameters, the linear regression was also conducted on their log transforms to determine which provided the more normal set of residuals.

For each multiple linear regression equation, the linearity of the relationship between the dependent and independent variables, and the normality and homoscedasticity of the error terms were evaluated.

To assess linearity, a graphical method was used to determine whether the response appeared linear to each of the covariates in turn that remained within the regression. This was performed by correcting the CCP parameter using the other covariates and factors to those for the International Radiation Protection Commission's standard man (age 25 years, height 1.8 m, mass 70 kg) (ICRP, 1975) and then plotting these values against the remaining covariate. Should the relationship not appear linear, then other forms for the covariate could be explored, for example a polynomial, and the significance of any improved fit assessed using Akaike's information criterion (Akaike, 1974).

These results illustrate whether and how each of the four CCP parameters are influenced by the participant's age, height, BMI and sex.

In order to provide initial, preliminary estimates for the normal ranges associated with the CCP parameters, the variability of individuals around their predicted values from Equation (1) was studied. Normality of the residuals was assessed using the Shapiro–Wilk (Shapiro & Wilk, 1965) and Kolmogorov–Smirnov (Massey Jr, 1951) tests. For each CCP parameter, any outlying values from the regression were checked to determine whether there was any reason the value should be excluded. If so, the regression was repeated with those data points excluded. Box–Cox transforms (Box & Cox, 1964) were then employed to determine whether there were any transforms of the dependent variable in the regression that gave residuals that were significantly closer to normal than those obtained using the untransformed, or log‐transformed variable.

Homoscedasticity was assessed using the Breusch–Pagan (Breusch & Pagan, 1979) and modified Breusch–Pagan (Koenker, 1981) tests to determine whether the variance of the errors from the regression was dependent on the values of the predictors. Provided this was not the case, then the root of the mean squared error could be used to provide a standard deviation for the normal range. On the other hand, if significant heteroscedasticity were present, then further modelling of the random effects would be required.

Once final regression equations had been obtained for each of the CCP parameters, *z*‐scores for each could be calculated as:

(2) $$z=\left(y_{\mathrm{obs}}-y_{\mathrm{pred}}\right)/\sigma_{y},$$

where *y*_obs_ is the observed CCP parameter value (or its transform), *y*_pred_ is the predicted CCP parameter value or its transform and σ*_y_* is the root mean squared error from the regression. By way of an initial exploration of these *z*‐scores in disease, values were calculated for data from early CCP studies in asthma, COPD and cystic fibrosis (Sandhu et al., 2023; Smith et al., 2020, 2022). For this exploration, the upper and lower limits of normal were taken to correspond to the 95th and 5th percentile, respectively, which equate to *z*‐scores of ±1.65.

## 3 RESULTS

### 3.1 Participants

Of the 106 original participants, 12 were removed from the analysis because they had an absolute N_2_ imbalance >60 mL/min during the airbreathing phase of the test. A further two participants were excluded later in the analysis (see ‘Prediction equations’, below), which left a total of 92 participants whose data were used in the final analysis. A little over half the participants (54) had undergone spirometry with mean values for forced expired volume in one second (FEV_1_) of 97.4 ± 16.7% predicted, for forced vital capacity (FVC) of 100.2 ± 15.7% predicted and FEV1/FVC of 0.795 ± 0.059. The participants’ physical characteristics are shown in Table 2.

The raw data, including CCP parameter values and the associated z‐scores, can be found in the supporting information.

**TABLE 2:** Participant characteristics.

Characteristics	Normal control cohort		
	Male	Female	Overall
Number of participants	67	25	92
Age / years	39.3 ± 18.4 (19–80)	40.2 ± 18.9 (19–80)	39.6 ± 18.6 (19–80)
Height / m	1.79 ± 0.07 (1.58–1.94)	1.65 ± 0.07 (1.58–1.80)	1.75 ± 0.09 (1.58–1.94)
Weight / kg	78.4 ± 11.3 (59.0–113.0)	60.7 ± 10.4 (45.0–82.5)	73.6 ± 13.6 (45.0–113.0)
BMI / kg m^−2^	24.5 ± 3.6 (19.6–40.5)	22.2 ± 3.7 (16.6–29.8)	23.9 ± 3.8 (16.6–40.5)

Abbreviations: Data are means ± SD (range). BMI, body mass index.

### 3.2 CCP modelling

The mean (±SD) values returned across the 92 participants for the four CCP parameters for which normal ranges were to be constructed as functions of the participants’ physical characteristics were as follows: FRC, 3.33 ± 0.75 L; *V* d, 0.150 ± 0.040 L; σ*V* d, 0.35 ± 0.06; and σlnCl, 0.51 ± 0.09. The mean values for the other parameters estimated by CCP were as follows: the fractional expansion of the deadspace volume relative to the fractional expansion of the alveolar volume (Cvd), 0.61 ± 0.37; the air‐breathing oxygen consumption (${\dot{V}}_{O_{2}}$), 0.298 ± 0.055 L min^−1^; the respiratory quotient (*R*), 0.81 ± 0.06; and the ideal $P_{{\mathrm{CO}}_{2}}$ (${\mathrm{Pi}}_{{\mathrm{CO}}_{2}}$), 5.00 ± 0.45 kPa. These other parameters were not further studied.

### 3.3 Prediction equations

The regression analysis was initially undertaken in 94 participants for FRC, *V* d, σ*V* d and σlnCl and for the log‐transform of these variables.

For each variable, outlying values were checked to determine whether there was any reason why they should not be included within the dataset. For one participant, there was a very large value for FRC that appeared to have resulted from a small N_2_ leak. For another (young) participant, three of the four variables were very significant outliers, and we concluded that this participant most likely had unrecognised lung disease. These two participants were removed from the analysis, but all other outliers were retained.

Following these two exclusions, the regression analysis was repeated on the 92 remaining participants. Non‐significant predictors were removed sequentially, least significant first, until only the significant predictors remained. For each CCP parameter, the quality of fit was inspected visually against each of the retained predictors. This was achieved by using the regression coefficients to adjust each data point to reflect the physical characteristics of a standard human for all predictors apart from the one under consideration. For FRC, *V* d and σ*V* d, visual inspection suggested that the regression equations described the data satisfactorily. However, the response of σlnCl against its single predictor of ln(age) did not appear linear because there was little change in the values between 20 and 30 years of age. To address this, a second predictor of (ln(age))^2^ was added, and this significantly improved the fit as judged by a reduction in Akaike's information criterion.

**FIGURE 3:**
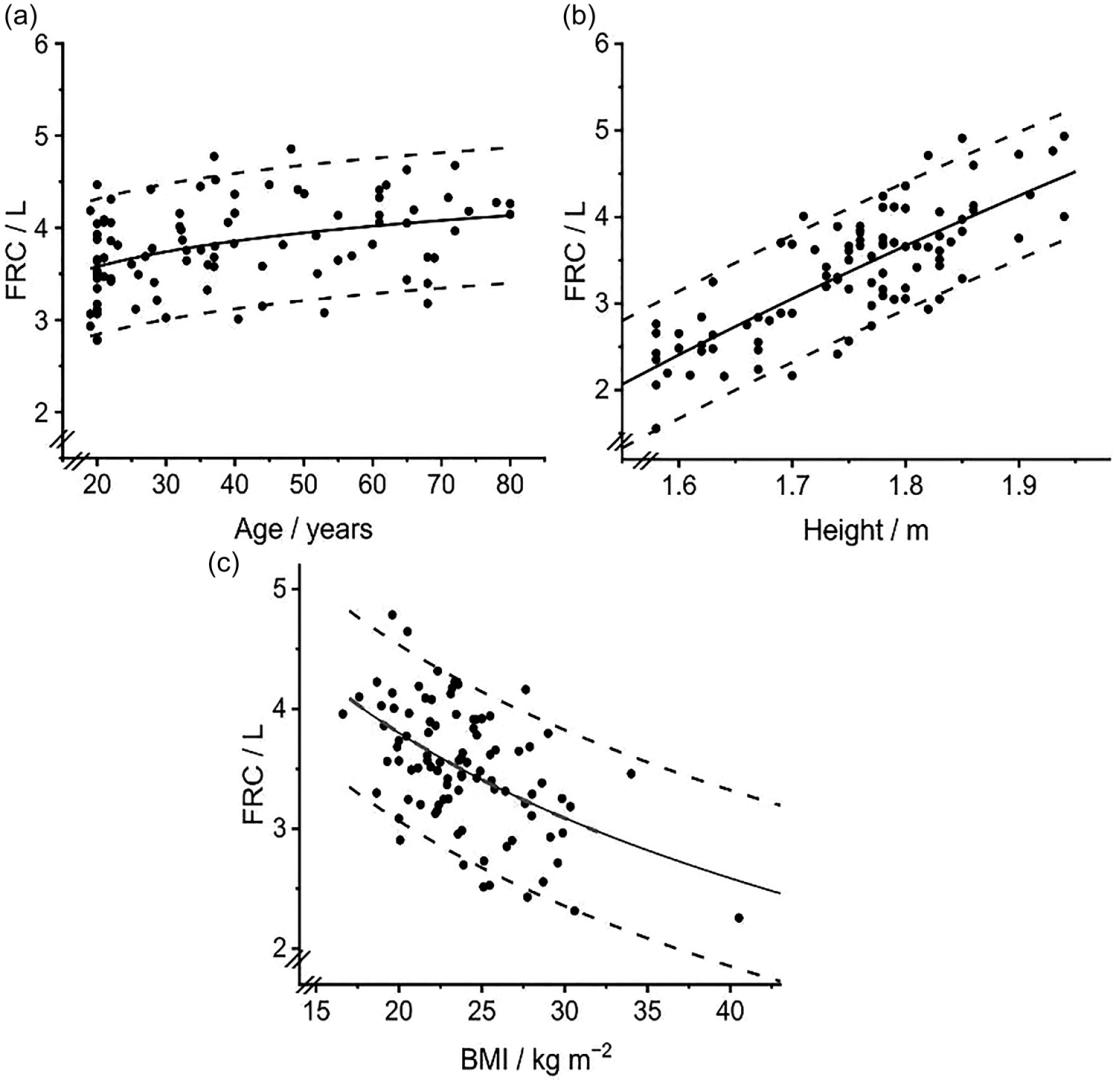
Variation in functional residual capacity (FRC) with age (a), height (b) and body mass index (BMI) (c). Data points (*n* = 92) illustrate values for individuals corrected to that for standard man (height 1.8 m, age 25 years and BMI 21.6 kg m^−2^), except in the case of the variable represented on the abscissa. Continuous line indicates predicted value. The effect of excluding the two participants with the highest BMI on the prediction is illustrated in (c) by a second (black dashed) line over the BMI range of 15–32, but this is mostly obscured by the original prediction. The other dashed lines are the 5th and 95th percentile.

**FIGURE 4:**
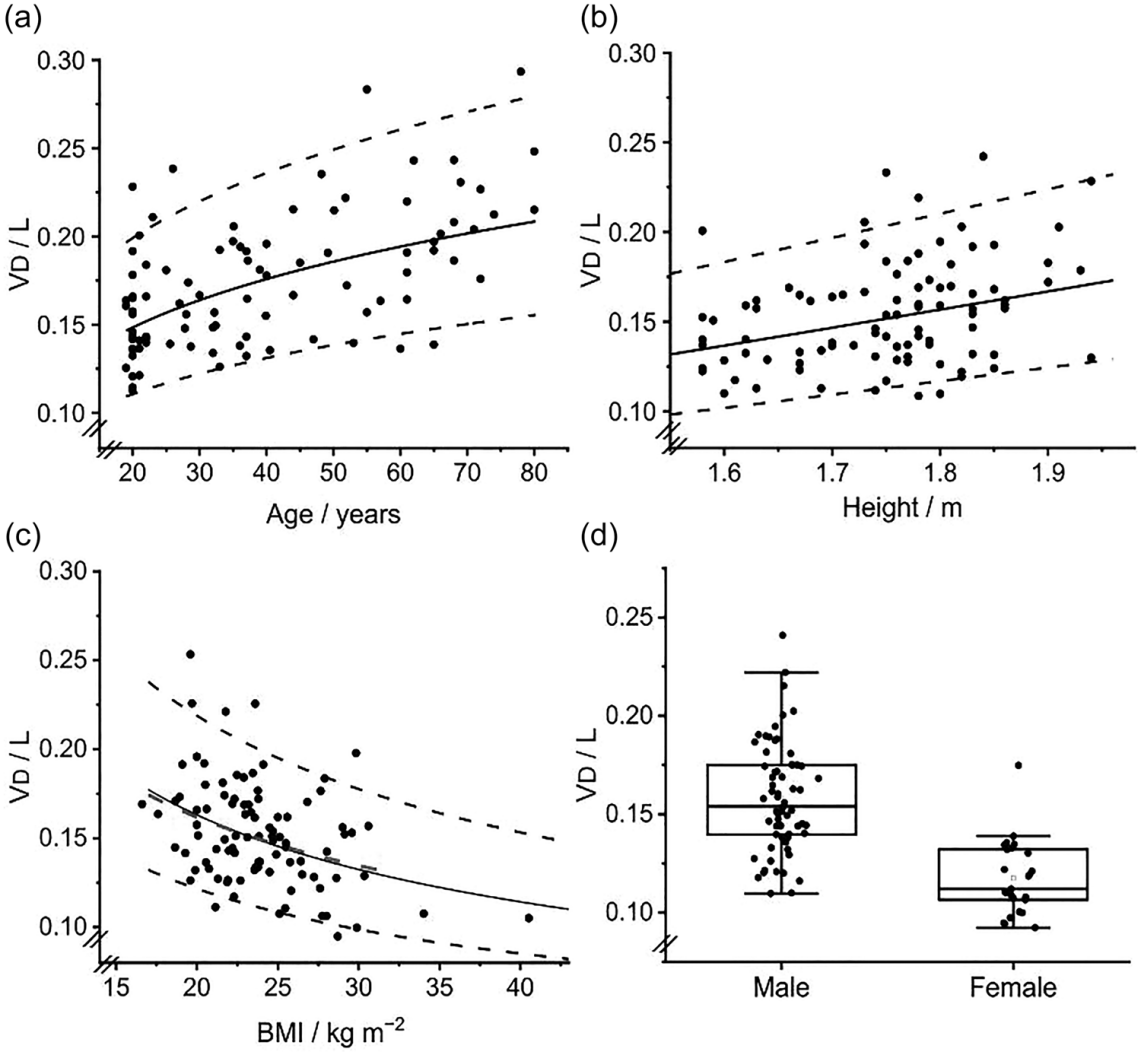
Variation in anatomical dead space (*V* d) with age (a), height (b), body mass index (BMI) (c) and sex (d). Data points (*n* = 92) illustrate values for individuals corrected to that for standard man (male, height 1.8 m, age 25 years and BMI 21.6 kg m^−2^), except in the case of the variable represented on the abscissa. Continuous line indicates predicted value. The effect of excluding the two participants with the highest BMI on the prediction is illustrated in (c) by a second (black dashed) line over the BMI range of 15–32, but this is mostly obscured by the original prediction. The other dashed lines are the 5th and 95th percentile. For box plots, the box illustrates the median, 25th and 75th centiles, and the whiskers illustrate the maximum and minimum values within 1.5 times the interquartile range.

**FIGURE 5:**
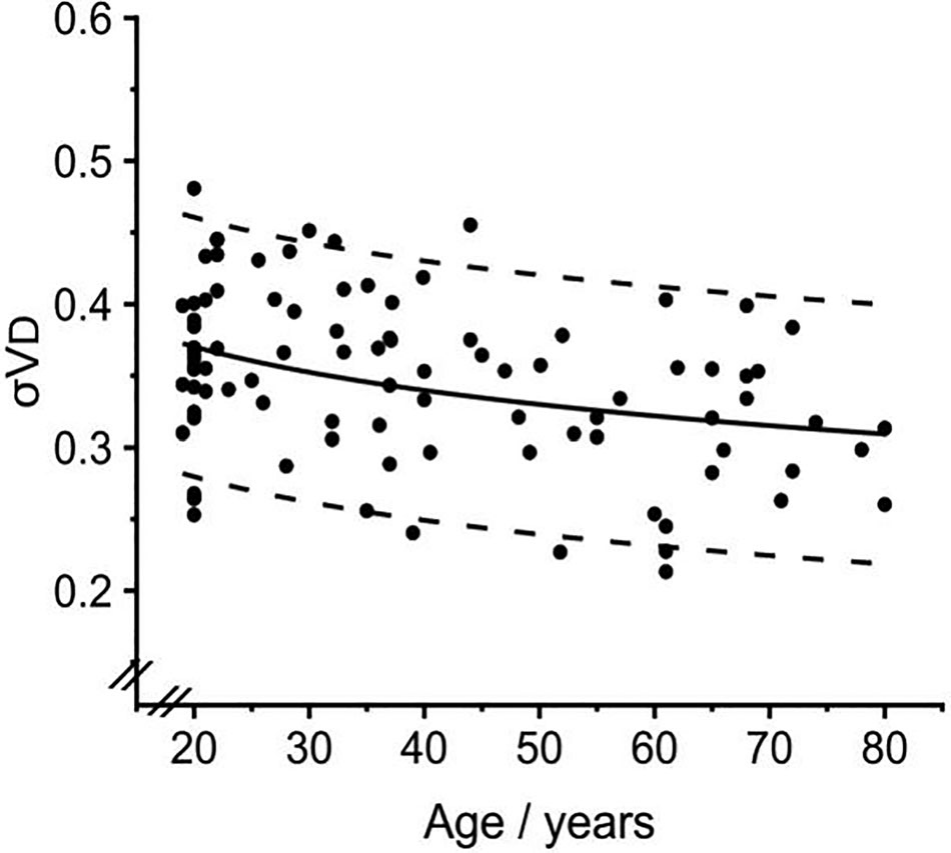
Variation in the standard deviation for the standardised dead space (σ*V* d) with age. Continuous line indicates predicted value. Dashed lines are the 5th and 95th percentile. Data for 92 individuals.

**FIGURE 6:**
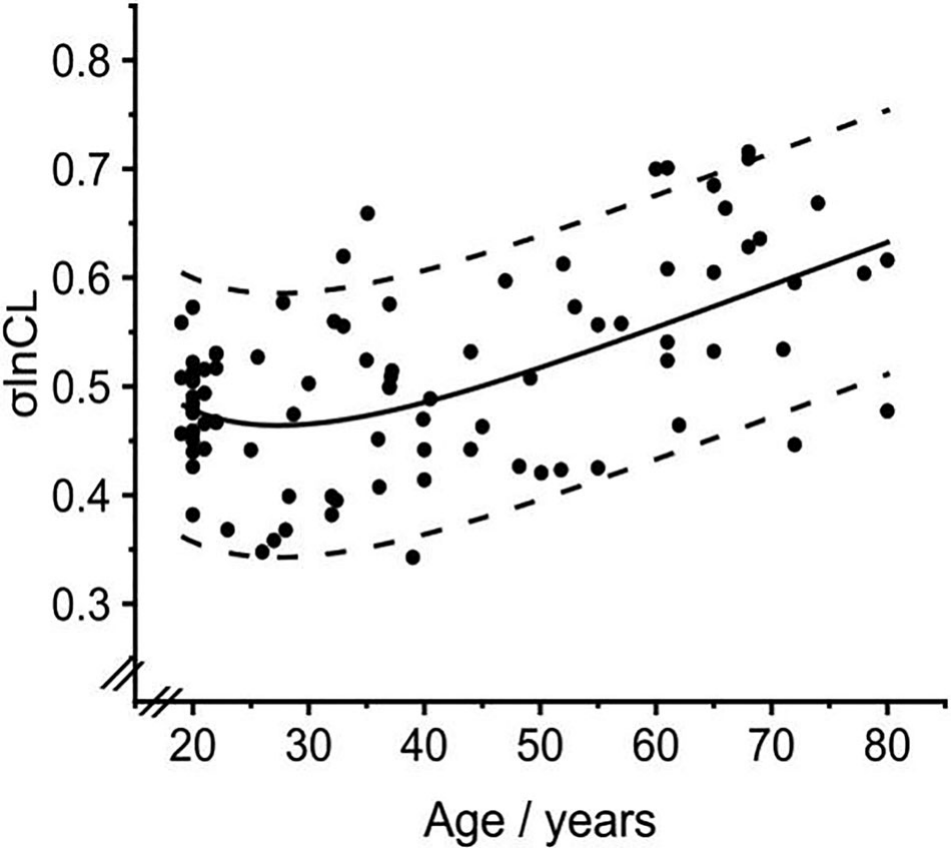
Variation in the standard deviation of the natural logarithm for the standardised lung compliance (σlnCl) with age. Continuous line indicates predicted value. Dashed lines are the 5th and 95th percentile. Data for 92 individuals.

Values for the two volume‐related parameters, FRC and *V* d, both increased significantly with increasing age and height, and decreased significantly with increasing BMI. Values for *V* d, but not FRC, were significantly lower for females than males (after controlling for age, height and BMI in the regression). In contrast, the values for the two inhomogeneity‐related parameters, σ*V* d and σlnCl, were only significantly affected by the participants’ age, and not by any other of the physical characteristics studied. The regression coefficients together with their respective *P*‐values are given in Table 3. The associated curvilinear relations that arise when the predictors are plotted on a linear scale are illustrated in Figures 3, 4, 5, 6 for the chosen transforms (see paragraph below) for FRC, *V* d, σ*V* d and σlnCl, respectively.

**TABLE 3:** Regression coefficients.

Parameter		Constant	ln(age)	(ln(age))^2^	ln(height)	ln(BMI)	is_female	√MSE
FRC / L	Coefficient	1.462	0.401	—	10.699	−1.749	—	0.4467
	*P*	0.182	<0.001	—	<0.001	<0.001	—	
ln(*V* d)	Coefficient	−1.746	0.245	—	1.163	−0.514	−0.290	0.1787
	*P*	<0.001	<0.001	—	0.018	<0.001	< 0.001	
σ*V* d	Coefficient	0.502	−0.044	—	—	—	—	0.055
	*P*	<0.001	<0.001	—	—	—	—	
σlnCl	Coefficient	2.062	−0.966	0.146	—	—	—	0.0738
	*P*	<0.001	0.004	0.002	—	—	—	

Abbreviations: BMI, body mass index; FRC, functional residual capacity; ln(*V* d), natural logarithm of the anatomical dead space (end‐inspiratory); σlnCl, SD for the natural logarithm for the standardised lung compliance; σ*V* d, SD for the standardised dead space; √(MSE), square root of the mean squared error.

Two participants had values for BMI that were considerably higher than for the other participants. To explore the significance of this, a sensitivity analysis was performed where the regression analyses were repeated with these two participants excluded. This did not affect the significance or otherwise of any predictor for any of the CCP parameters. For the two parameters for which BMI was significant (FRC and *V* d), the effect on the regression relationship was minimal (Figures 3c and 4c), and the effect on the square root of the mean squared error was less than 1% in each case.

**FIGURE 7:**
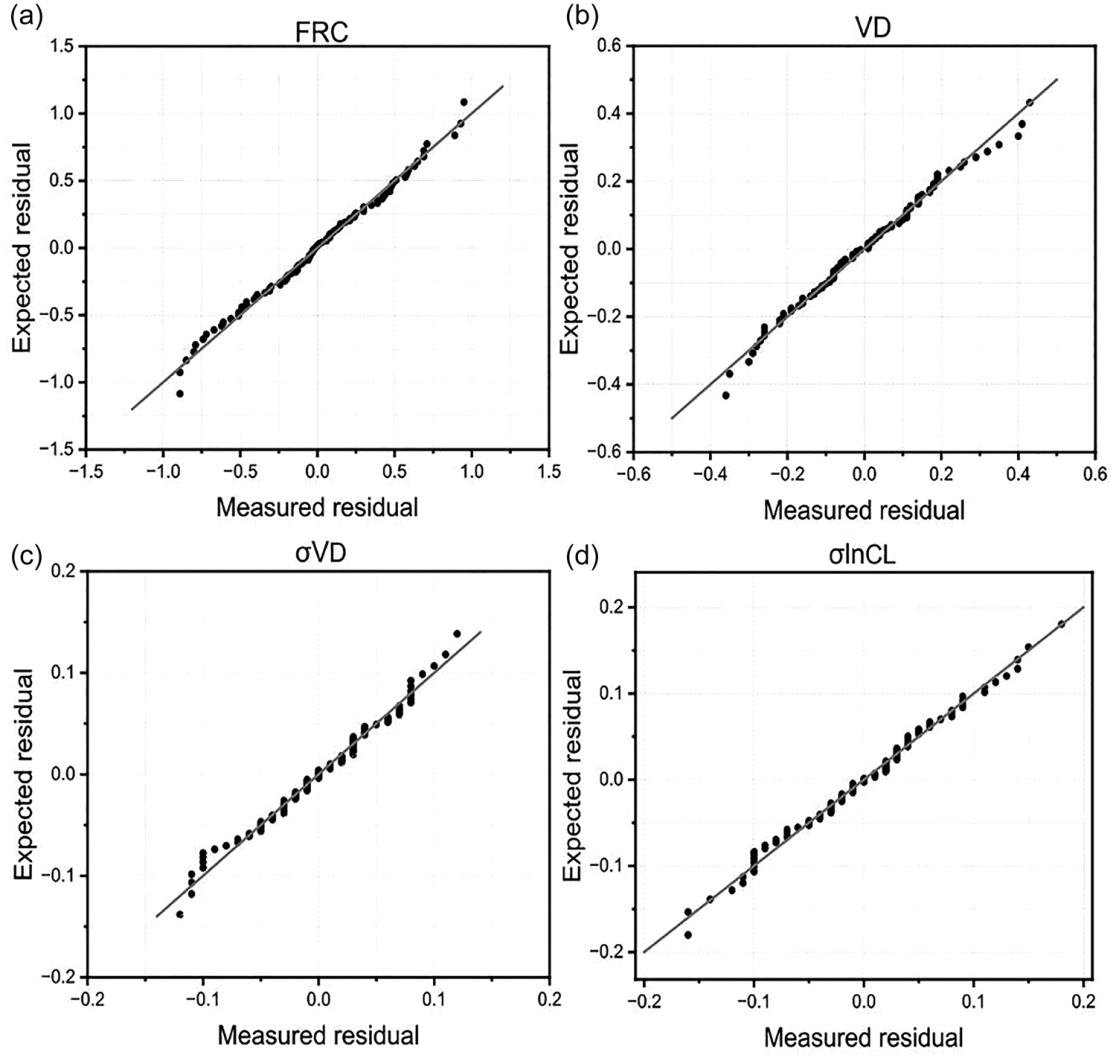
Standardised Q‐Q plots for expected residual against the residual from the regression. (a) Functional residual capacity (FRC); (b) anatomical dead space (*V* d); (c) standard deviation (SD) for the standardised dead space (σ*V* d); (d) SD for the natural logarithm for the standardised lung compliance (σlnCl). Data for 92 individuals.

The normality of the residuals was assessed both graphically through Q‐Q plots and through the Kolmogorov–Smirnov and Shapiro–Wilk tests. On the basis of these results, a log transform was used for *V* d as giving the more normally distributed residuals, but the other three variables (FRC, σ*V* d, σlnCl) were left untransformed. As an additional check, Box–Cox transforms were used for the dependent variable to identify the power law (λ) that resulted in residuals that were closest to normal. In each case, the confidence interval for lambda included the value associated with the simple transform (λ = 0 for *V* d, λ = 1 for FRC, σ*V* d, σlnCl). Values for the Kolmogorov–Smirnov and Shapiro–Wilk tests for the normality of the residuals together with their respective *P*‐values are shown in Table 4. The Q‐Q plots for the regressions are shown in Figure 7.

**TABLE 4:** Normality and heteroscedasticity tests of the data for the 92 participants.

Parameter		Shapiro–Wilk	Kolmogorov–Smirnov	Modified Breusch–Pagan	Breusch–Pagan
FRC / L	Statistic	0.987	0.039	0.606	0.442
	*P*	0.532	0.200^†^	0.436	0.506
Ln(*V* d)	Statistic	0.988	0.046	1.772	1.520
	*P*	0.588	0.200^†^	0.183	0.218
σ*V* d	Statistic	0.983	0.048	0.667	0.494
	*P*	0.260	0.200^†^	0.414	0.482
σlnCl	Statistic	0.971	0.080	6.321	4.173
	*P*	0.040	0.190	0.012	0.041
σlnCl ^*^	Statistic	0.992	0.047	3.448	2.837
	*P*	0.848	0.200^†^	0.063	0.092

^†^Lower bound for significance. Abbreviations: FRC, functional residual capacity; σlnCl, SD for the natural logarithm for the standardised lung compliance; σlnCl ^*^, refitted σlnCl with inclusion of (ln(age))^2^ as a predictor in the regression; σ*V* d, SD for the standardised dead space; *V* d, anatomical dead space (end‐inspiratory).

### 3.4 *z*‐Scores

**FIGURE 8:**
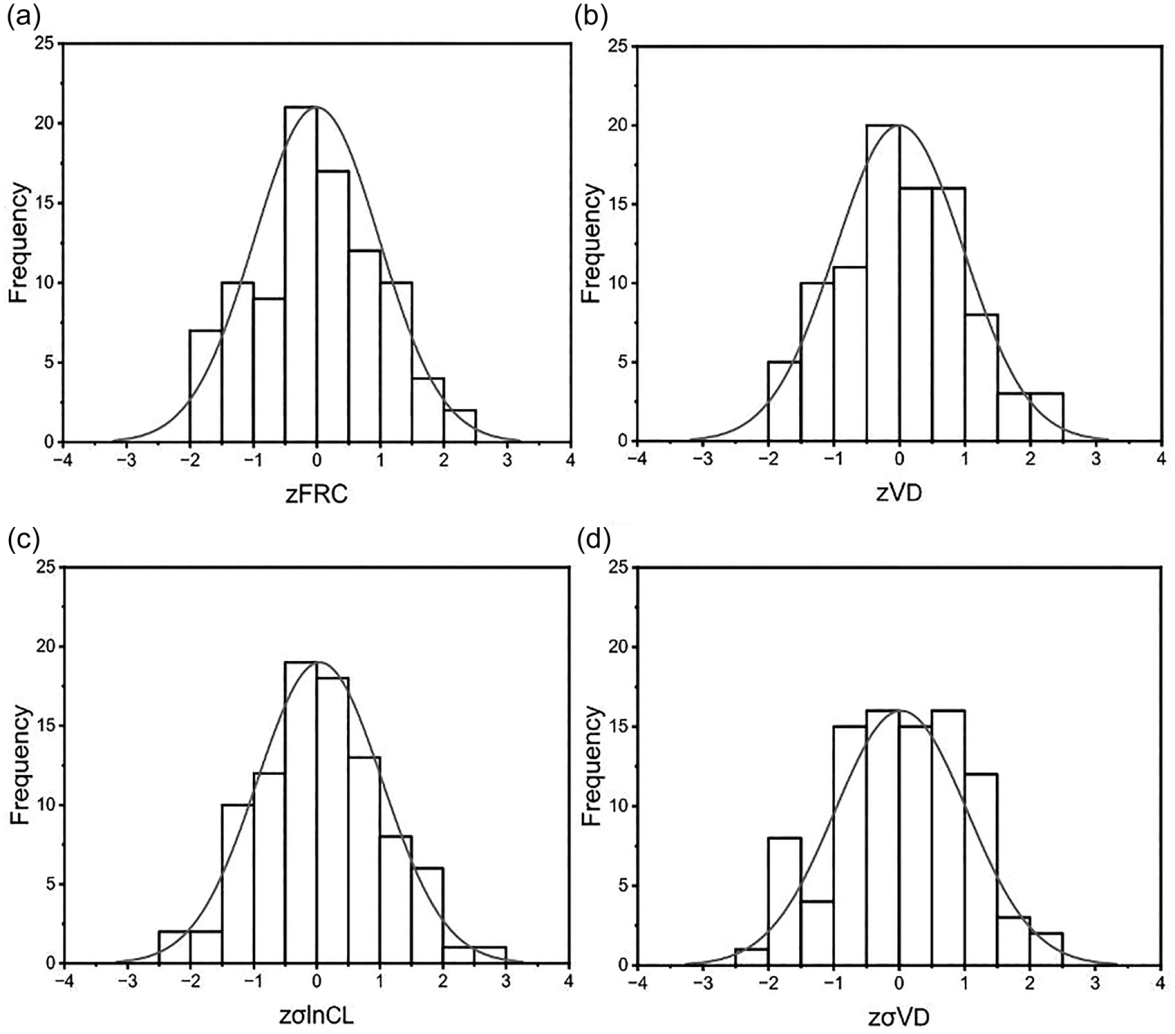
Histograms of the *z*‐scores for the heathy participants. (a) Functional residual capacity (zFRC); (b) anatomical dead space (z*V* d); (c) standard deviation (SD) for the natural logarithm for the standardised lung compliance (zσlnCl); (d) SD for the standardised dead space (zσ*V* d). Curves indicate the standard normal distribution. Data for 92 individuals.

The results from the Breusch–Pagan and modified Breusch–Pagan tests used to determine whether the residuals varied in size with the predictors are shown in Table 4 together with their respective *P*‐values. For all variables, no heteroscedasticity was detected and consequently the mean squared error from the regression can be used directly as an estimate of the variance. These estimates for the variance, when combined with the predicted value for each participant, allow each measurement to be transformed into a *z*‐score. Histograms illustrating the distribution of *z*‐scores for the participants are illustrated in Figure 8. For each parameter, these distributions appear to correspond fairly closely to the standard normal distribution.

**FIGURE 9:**
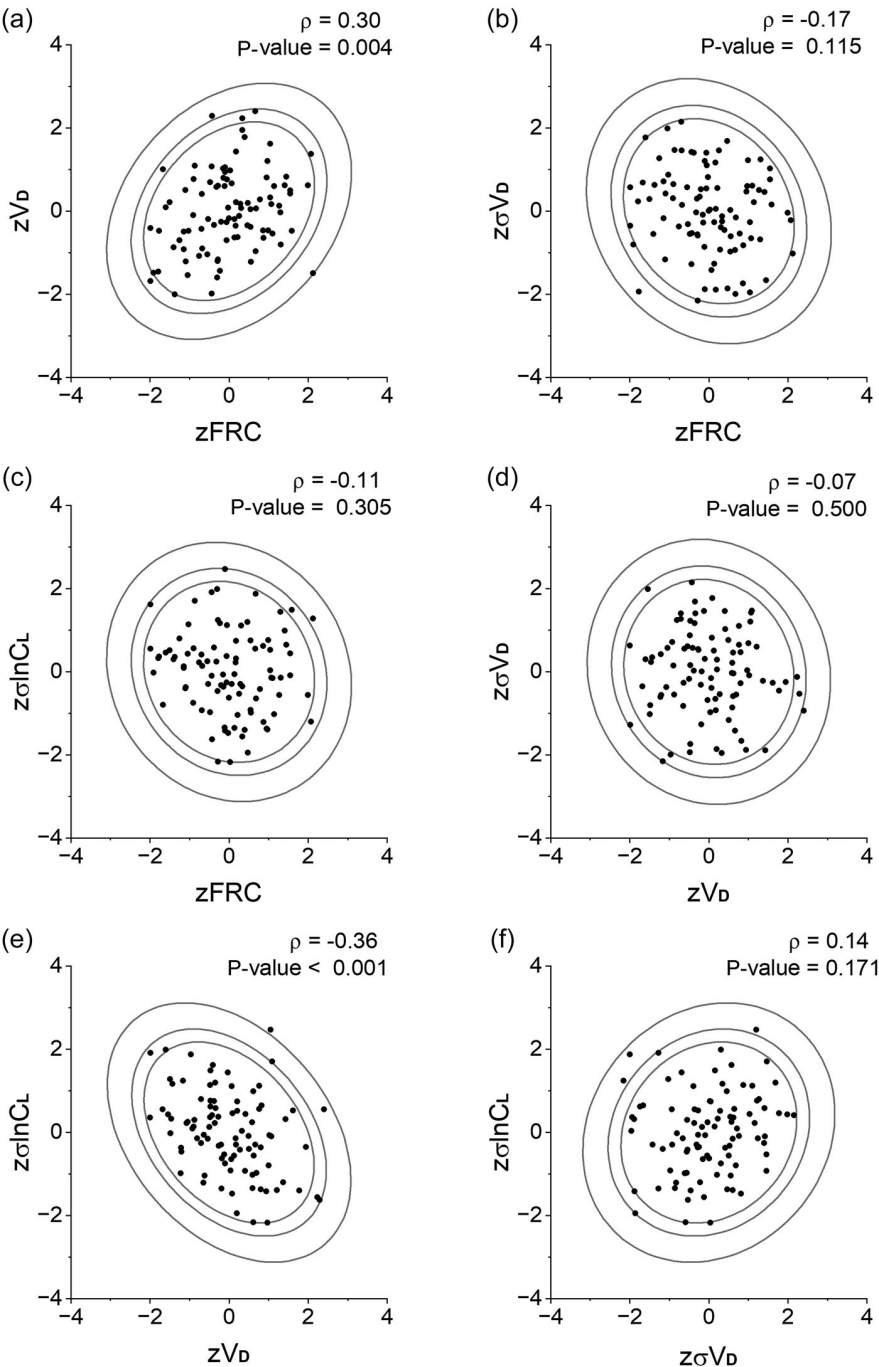
Correlations between *z*‐scores for different pairs of variables. (a) *zV* d vs. *z*FRC. (b) *z*σ*V* d vs. *z*FRC. (c) zσlnCl vs. *z*FRC. (d) *z*σ*V* d vs. *zV* d. (e) *z*σlnCl vs. *zV* d. (f) *z*σlnCl vs. *z*σ*V* d. Elliptic contours (inner to outer) predicted to contain 90%, 95% and 99% of all values, respectively. Data for 92 individuals.

The correlations between *z*‐scores for different pairs of parameter values are illustrated in Figure 9 together with their Pearson correlation coefficients and their associated *P*‐values. The use of *z*‐scores should remove any correlation between CCP parameters that arises simply because of differences in physical characteristics between participants. Two of the correlations were significant, and the other four were non‐significant. However, for none of these variables was the correlation large.

**FIGURE 10:**
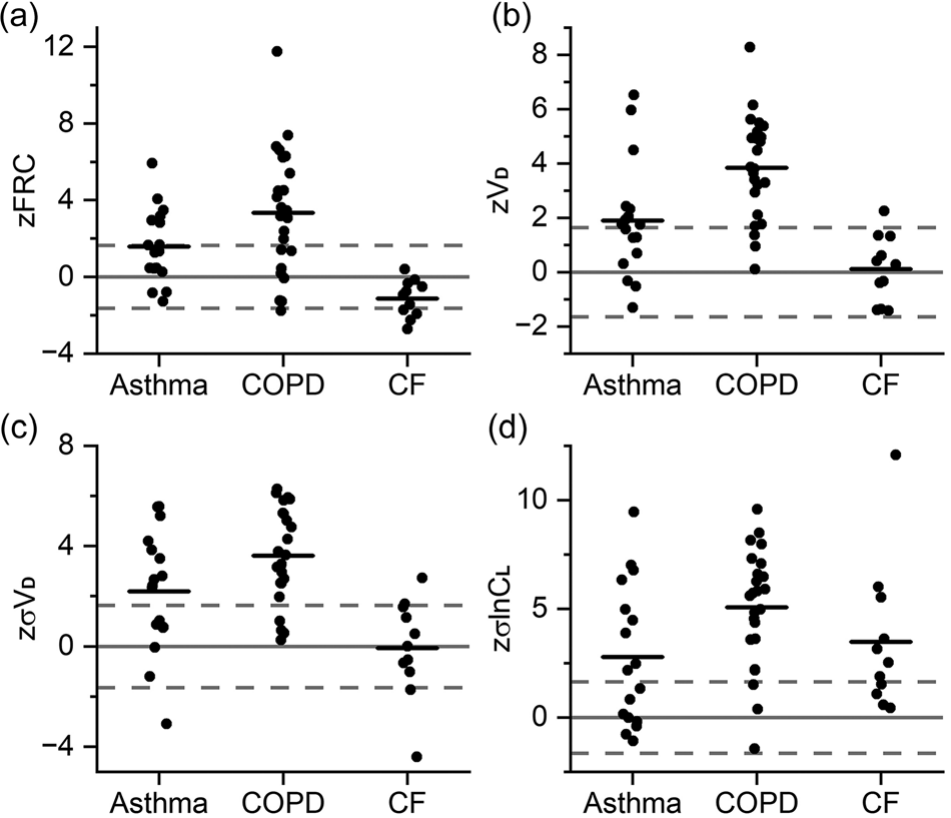
Z‐scores for model parameters for patients with asthma, chronic obstructive pulmonary disease (COPD) and cystic fibrosis (CF). (a) Functional residual capacity (zFRC); (b) anatomical dead space (z*V* d); (c) standard deviation (SD) for the standardised dead space (zσ*V* d); (d) SD for the natural logarithm for the standardised lung compliance (zσlnCl). Broken lines illustrate the 5th and 95th centiles at *z*‐scores of ±1.65. Numbers of patients: asthma, *n* = 17; COPD, *n* = 24; and CF, *n* = 11.

Figure 10 illustrates the *z*‐scores for FRC, *V* d, σVd and σlnCl calculated from three small, previously published, studies of patients with asthma, COPD and cystic fibrosis (Sandhu et al., 2023; Smith et al., 2020, 2022). The asthma study recruited an otherwise unselected group of patients with asthma attending a tertiary referral clinic for their disease, the COPD study recruited patients with differing severities of disease and the cystic fibrosis study focused just on patients with early stage disease and a preserved forced expiratory flow in one second (FEV_1_ % predicted). Values outside the normal range are present for many of the patients. Mostly, values for FRC, *V* d, σ*V* d and σlnCl appear overly large in the disease states, although for cystic fibrosis some values for FRC are below the proposed lower limit of normal.

## 4 DISCUSSION

Like so many chronic conditions, chronic airways disease causes progressive and irreversible tissue remodelling, in this case in the lung. Thus, when trying either to understand the pathophysiology or to improve outcomes, it is important to be able to identify the presence of disease as soon as possible. Standard clinical measurements of lung function, such as FEV_1_ % predicted, perform poorly in this regard, in part because they represent mean overall values in the lung and these take time and a significant amount of disease to change, and in part because the range of normal variation is large. In early disease, simple probability dictates that the distribution of disease in an organ should be patchy or uneven. Thus, measurements that detect inhomogeneity (unevenness) across the lung – that is, variance‐based measures of lung properties – may well detect disease far sooner than measurements that simply reflect mean properties like FEV_1_. One of the main goals of CCP is to recover variance‐based measures of lung function, such as the measures of inhomogeneity, σ*V* d and σlnCl. The present study, as well as examining how selected CCP parameters are affected by participants’ physical characteristics, also develops an initial approach, through the use of *z*‐scores, to determine whether values for CCP parameters lie within a normal range (here defined as between the 5th to 95th centiles) that could potentially be used to detect early lung disease.

Among the four parameters identified by CCP that have been studied here, FRC is the only one that is measured and reported as part of a standard clinical lung function test. Thus, for this variable, it is already well understood as to how it will vary with physical characteristics, and thus provides a means of comparing our findings with those from much larger datasets. If we enter the age, sex and height of each of our 92 participants into the Global Lung Initiative's (GLI) (Hall et al., 2021) established set of prediction equations that were developed using a modern, non‐parametric approach (Rigby & Stasinopoulos, 2004), then the average predicted value for FRC was just 1.7% (55 mL) below the average of either our measured or predicted values. The parameters of the GLI prediction equations cannot be compared precisely with the parameters of our equations because the equations are different. GLI predicts the log of the median FRC, it uses separate equations for males and females, the equations do not include BMI and they have an additional spline term. However, after undertaking an approximate correction for the use or otherwise of a log term for FRC, the coefficients for the predictors of log(height) and log(age) were both quite similar between GLI and the present study (within 20–30% of each other). Although GLI did not use BMI in their prediction equations, it is well recognised that BMI has a major influence on FRC (Jones & Nzekwu, 2006). The form of the relationship given by Jones & Nzekwu (2006) is an exponential decline in FRC with increasing BMI which appears similar to our Figure 3. Indeed, in both Jones & Nzekwu (2006) and our study, the decline in FRC between BMI values of 20 and 30 was ∼25%. Overall, these comparisons suggest that the results drawn from our sample of 92 participants reasonably reflect those obtained using a much larger dataset (the GLI findings were drawn from 7190 observations from 17 centres).

Unlike FRC, there are no large‐scale datasets available for *V* d. However, it is worth noting that the signs for all the predictors (age, height, BMI, sex) are the same for *V* d as they are for FRC (although sex did not reach significance for FRC), suggesting that there are similar influences on the two volumes. Fowler's classic study (Fowler, 1948) of deadspace provided an average value of 0.156 L for 45 males and 0.104 L for four females. These values compare very well with our predictions of 0.155 L and 0.115 L obtained by using the average age, height and weight for Fowler's male and female participants, respectively. Fowler also noted that *V* d could not really be treated as a single volume because it increased with increasing tidal volume, becoming ∼0.1 L greater during forced breathing at maximal tidal volume. This is potentially problematic because participants who are naïve to breathing on a mouthpiece often breathe more slowly and with larger tidal volumes than normal. In order to control for this, we estimated an auxiliary non‐dimensional parameter within the CCP model, Cvd, which was bounded at 0 for a rigid deadspace and at 1 for a deadspace that expanded in proportion to alveolar expansion. We then used this to adjust the deadspace to a value appropriate for a tidal volume associated with a respiratory rate of 13 breaths per minute at the participant's metabolic rate while keeping their arterial $P_{CO_{2}}$ as before. One limitation of this approach was that the free‐breathing protocol used is not necessarily a very good protocol for estimating Cvd, particularly when a participant breathed very regularly with little variation in tidal volume.

The parameter σ*V* d is unique to the CCP approach and so there are no comparisons to be drawn with the rest of the literature. Apart from age, there were no significant effects of any of the physical characteristics of the participants on this variable. As the sign of the regression coefficient for age is the opposite of that for ln*V* d, as a participant ages, the value for σ*V* d should fall as the value for ln*V* d rises. In the CCP model, σ*V* d interacts with *V* d so as to determine the width of the distribution for the different deadspace pathlengths in terms of absolute volume, and so the width of this distribution in terms of absolute volume should vary less with ageing than does the total volume (*V* d) itself. Finally, it should be noted that σ*V* d is a functional measure, and so includes the effects of dispersion within the airways as well as differences in anatomical pathlength.

As for σ*V* d, the parameter σlnCl is unique to the CCP approach, and again it showed no significant variation with any of the physical characteristics apart from age. In contrast to the other parameters, the relationship of σlnCl with age was relatively flat between the ages of 20 and 30 before it began to rise with increasing age. This finding is also characteristic (albeit inverted) for the change in FEV_1_ with ageing, and indeed the GLI commented on the differences between the spirometric and volumetric parameters over this time interval in their datasets (Hall et al., 2021), which perhaps reflect differences between the completion of lung maturation and the onset of lung function decline.

As well as exploring how the CCP parameters vary with physical characteristics, this study also sought to provide some initial, very preliminary, estimates for their normal ranges. As we were limited to ∼90 observations, the approach adopted was necessarily a parametric one. We used linear regression, selected a transform for each variable that resulted in residuals that did not differ significantly from normal (so trying to avoid both skewness and differences between the median and the mean), and then checked for heteroscedasticity (which, if not present indicates that there was not a significant variation of the coefficient of variation with the predictors). This dependence on the normal distribution is less than ideal, but necessary given the limited number of observations. Nevertheless, for both the inhomogeneity terms (σ*V* d and σlnCl), the only physical characteristic that had a significant influence on their values was age, there was no evidence of heteroscedasticity in relation to this predictor, and furthermore the residuals appeared close to normal. These features all should help to provide better estimates for the normal ranges from this number of datasets than would otherwise be the case.

The development of normal ranges allowed the calculation of *z*‐scores for data from three pre‐existing small datasets for asthma, COPD and cystic fibrosis, and these values were abnormal in many patients. Of these patients, those with COPD would have undergone the most airway remodelling and lung destruction, and all but four of them had an abnormal FEV_1_ (<80% predicted). In these patients, almost all *z*‐scores were abnormal for both inhomogeneity parameters, σ*V* d and σlnCl, and also for *V* d. In contrast, asthma is a disease defined primarily by variable airflow obstruction where there is a lesser degree of airway remodelling, and ∼50% of these patients had a normal FEV_1_ at the time of study. In these patients, a similar percentage had abnormal values for σ*V* d and σlnCl, a surprising percentage had abnormally elevated values for FRC, and a few patients had very high values for *V* d. Further study in a larger cohort is clearly warranted to understand to what degree these abnormalities are all associated with each other versus to what degree they reflect different, independent aspects of asthma. The patients with cystic fibrosis all had early disease and all had normal values for FEV_1_ (>80% predicted). Despite this, the majority of these patients had abnormal values for σlnCl. This finding does provide real encouragement that σlnCl may be a sensitive marker of early disease that could be useful for improving the understanding and management of airways disease. Interestingly, a proportion of these patients also appeared to have a low FRC, which perhaps reflects some effect of cystic fibrosis on the development and maturation within the parenchyma of the lung.

### 4.1 Study limitations

There are a number of limitations to the results presented here which should be borne in mind during interpretation. Of particular note, the study participants were not chosen as a carefully selected sample that would be representative of a wider general population. Indeed, many of the datasets used were collected as part of other, smaller studies, and this raises the question of how representative the results are likely to be for the general population as a whole. Some reassurance on this point can be gained by comparing standard measures of lung function for the participants with those expected for the wider population. However, only a subset of the individuals studied (54 out of 92) had undertaken spirometry. Furthermore, while the mean for the (GLI) predicted FRC values for our participants agreed very well with the mean of their measured values, the measured values were obtained through CCP rather than by using a standard clinical technique. For this comparison to carry more weight, there needs to be a robust validation between the CCP‐measured values and those determined with a standard clinical technique, such as body plethysmography. Future work to develop the prediction equations presented here would clearly benefit from standard lung function tests being undertaken on all participants to aid determining how representative participants are of the wider population. Finally, a larger sample size would enable an approach based on non‐parametric statistics to be adopted and so avoid any dependence on variables following a normal distribution.

### 4.2 Conclusions

In summary, this study reports how various CCP parameters are affected by individuals’ physical characteristics. It presents a preliminary set of reference equations for CCP parameters based on these findings and from which *z*‐scores can be calculated. Using the physical characteristics from our cohort, the mean overall difference between our predicted values for FRC and those made using the GLI equations was ∼1.7% of the mean (55 mL). For *V* d, our predicted values almost exactly matched the measured values from Fowler's pioneering work (Fowler, 1948) in this area. The closeness of our predictions for FRC and *V* d to other measurements made using different approaches on different cohorts is reassuring, and provides at least some encouragement that the predictions for σ*V* d and σlnCl, for which we have no comparators, may be similarly accurate.

## AUTHOR CONTRIBUTIONS

Conceived and designed research: Grant A. D. Ritchie, Nayia Petousi and Peter A. Robbins Performed experiments: Asma Alamoudi, Snapper Magor‐Elliott and Haopeng Xu Analysed data: Asma Alamoudi, Dominic Sandhu, Thomas D. E. Bithell, Nicholas M. J. Smith, Graham Richmond and Lorenzo S. Petralia Interpreted results of experiments: Grant A. D. Ritchie, Nayia Petousi and Peter A. Robbins Prepared figures: Asma Alamoudi and Nicholas M. J. Smith Drafted manuscript: Peter A. Robbins Edited and revised manuscript: Lorenzo S. Petralia, Nick P. Talbot, Grant A. D. Ritchie, Nayia Petousi and Peter A. Robbins. All authors have read and approved the final version of this manuscript and agree to be accountable for all aspects of the work in ensuring that questions related to the accuracy or integrity of any part of the work are appropriately investigated and resolved. All persons designated as authors qualify for authorship, and all those who qualify for authorship are listed.

## CONFLICT OF INTEREST

Oxford University Innovation, a wholly owned subsidiary of the University of Oxford, holds/has filed patents relating to the background IP for the technology. G.A.D.R. and P.A.R. have an interest in one or more patents.

## Supporting information



Individual participants' physical characteristics and CCP parameter values

## Data Availability

Data will be made available upon reasonable request.
